# Spatial Proteomics Using S4P

**DOI:** 10.21769/BioProtoc.5620

**Published:** 2026-03-05

**Authors:** Ritian Qin, Fuchu He, Weijie Qin

**Affiliations:** 1 School of Life Sciences, Tsinghua University, Beijing, China; 2 National Center for Protein Sciences (Beijing), Beijing Institute of Lifeomics, Beijing, China

**Keywords:** Spatial proteomics, Mass spectrometry, Sparse sampling, Deep learning, Image reconstruction

## Abstract

Spatial proteomics enables the mapping of protein distribution within tissues, which is crucial for understanding cellular functions in their native context. While spatial transcriptomics has seen rapid advancement, spatial proteomics faces challenges due to protein non-amplifiability and mass spectrometry sensitivity limitations. This protocol describes a sparse sampling strategy for spatial proteomics (S4P) that combines multi-angle tissue strip microdissection with deep learning–based image reconstruction. The method achieves whole-tissue slice coverage with significantly reduced sampling requirements, enabling mapping of over 9,000 proteins in mouse brain tissue at 525 μm resolution within 200 h of mass spectrometry time. Key advantages include reduced sample processing time, deep proteome coverage, and applicability to centimeter-sized tissue samples.

Key features

• Achieves whole-tissue slice coverage for spatial proteomics mapping.

• Enables reconstruction of spatial protein distribution using sparse sampling with multi-angle strip projections.

• Combines mass spectrometry–based proteomics with deep learning–based image reconstruction.

• Reduces required mass spectrometry time by 50%–90% compared to gridding-based approaches.

## Graphical overview


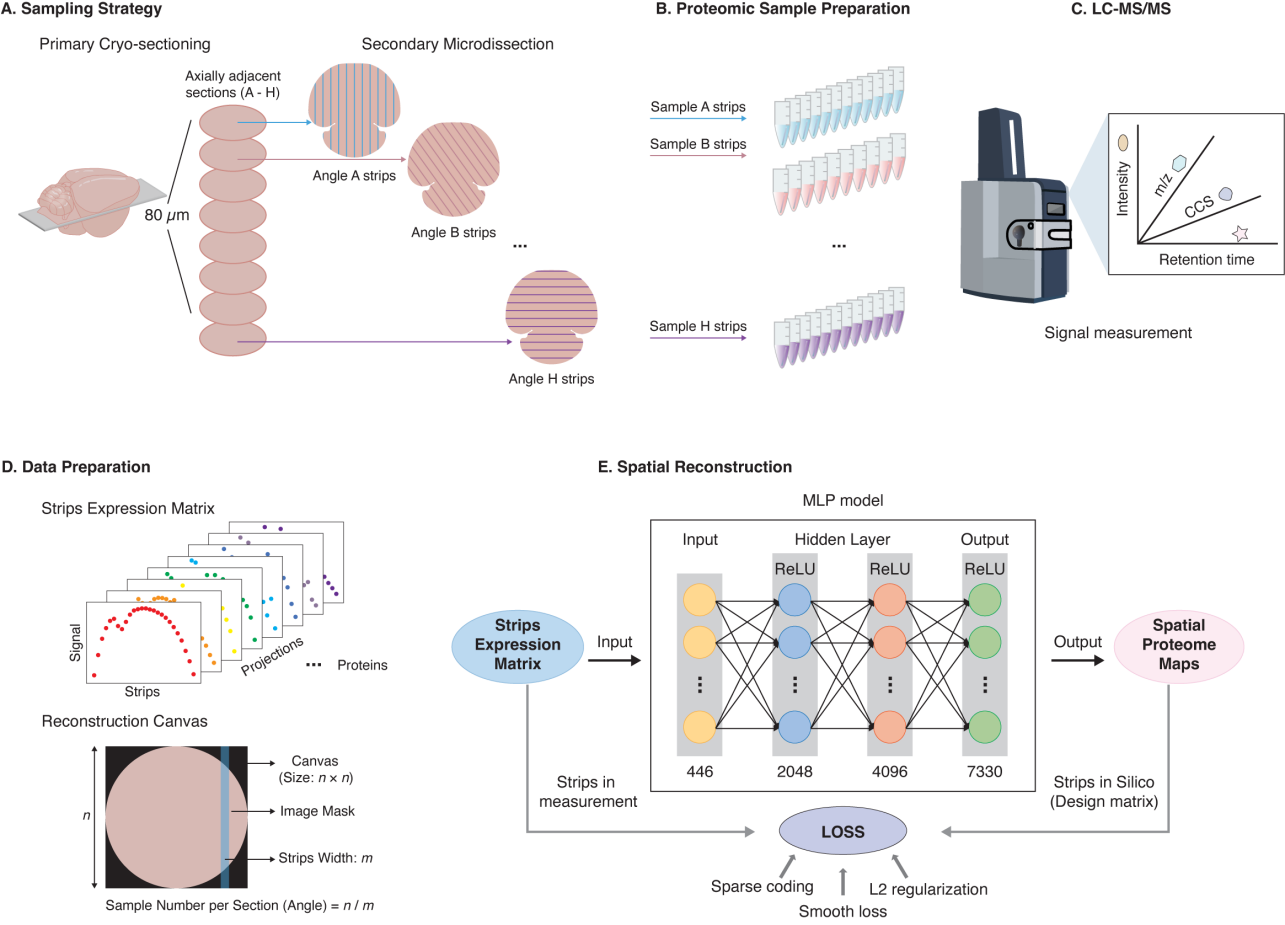


**Graphical overview of the sparse sampling strategy for spatial proteomics (S4P) method.** (A) Multi-angle strip microdissection from adjacent tissue sections. (B) Proteomic sample preparation of collected strips. (C) LC–MS/MS analysis of collected strips. (D) Data preparation for spatial reconstruction. (E) DeepS4P reconstruction of spatial protein maps.

## Background

Spatial mapping of biomolecules within tissues is essential for understanding cellular functions and interactions in their native context [1,2]. While spatial transcriptomics has advanced rapidly, enabling high-resolution and high-throughput mapping of gene expression [3–6], direct measurement of protein spatial distribution remains challenging. This discrepancy is critical given the often poor correlation between mRNA and protein abundance in mammalian tissues, which can lead to biased inferences about gene activity when relying solely on transcriptional data [7–11]. Existing spatial proteomics methods face significant limitations. Antibody-based techniques like imaging mass cytometry (IMC) offer single-cell resolution but are limited to analyzing only several dozen proteins [12]. Conversely, liquid chromatography–mass spectrometry (LC-MS) provides deep, untargeted proteome coverage but requires impractical amounts of instrument time for high-resolution whole-tissue mapping. For instance, mapping a 1-cm diameter tissue at 100 μm resolution using a “gridding” approach requires ~8,000 samples and over 8,000–10,000 h of MS time [13–15], making it infeasible for research applications.

The sparse sampling strategy for spatial proteomics (S4P) protocol presented here addresses these throughput and sensitivity bottlenecks. By integrating multi-angle tissue strip microdissection with a deep learning–based image reconstruction algorithm (DeepS4P), S4P significantly reduces the number of samples required for spatial reconstruction. This method enables the mapping of over 9,000 proteins in a mouse brain at 525 μm resolution within approximately 200 h of MS time, representing a 50% reduction compared to gridding-based strategies and potential reductions of 15–20 times for higher resolutions [13,14]. The primary advantages of S4P over existing methods include its ability to achieve whole-tissue slice coverage for centimeter-sized samples with deep proteome depth, while drastically reducing the formidable MS time previously required. A key limitation of the current protocol is its reliance on eight adjacent tissue slices for reconstruction, which provides an average profile across an 80 μm z-axis span. Furthermore, the minimum practical strip width is currently ~100 μm due to laser-induced tissue damage during microdissection. For future applications requiring single-cell resolution, S4P could be combined with tissue expansion methods [14] and microfluidic technology [16]. Beyond mapping protein distributions in complex organs like the brain, the S4P protocol is applicable to a wide range of tissues for investigating spatial proteome variations during development, disease progression, and drug treatment, offering a powerful tool for both basic research and translational studies.

While the S4P method represents a significant leap in whole-tissue, spatially resolved deep proteomics, it remains constrained by limited spatial resolution for analyzing subcellular compartments. To address this, the S4P framework can be enhanced through several promising avenues. First, improvements in spatial reconstruction algorithms can significantly boost the effective resolution derived from sparse sampling data. Second, integrating S4P with complementary techniques can bridge the resolution gap. For instance, combining S4P with methods like HyperLOPIT [17], which excels at assigning proteins to specific organelles through biochemical fractionation and multivariate analysis, could correlate tissue-scale architecture with detailed subcellular localization. Third, advanced sample preparation strategies can be employed, such as tissue expansion techniques [14] that physically enlarge specimens before S4P analysis, thereby achieving finer effective sampling. Finally, the workflow of S4P can be simplified and optimized through the adoption of microfluidic techniques [18] or robotic automation systems [19]. By combining these strategic improvements, S4P can evolve into a more versatile platform, offering a comprehensive, multi-scale framework for spatial proteomic investigation.

## Materials and reagents

**Biological materials**

1. Mouse tissue (C57BL/6J, 8-week-old female) (obtained from The Beijing Proteome Research Center)

2. Quality control: HeLa cells (ATCC, catalog number: CCL-2)

**Reagents**

1. Optimal cutting temperature compound (OCT) (Sakura Finetek, catalog number: 4583)

2. 2-methylbutane (isopentane) (Sigma-Aldrich, catalog number: M32631)

3. Urea (Sigma-Aldrich, catalog number: U1250)

4. Sodium deoxycholate (SDC) (Sigma-Aldrich, catalog number: D6750)

5. Tris-(2-carboxyethyl)-phosphine (TCEP) (Thermo Fisher Scientific, catalog number: 77720), freshly prepared

6. Chloroacetamide (CAA) (Sigma-Aldrich, catalog number: C0267)

7. Protease inhibitor cocktail (PIC) (Sigma-Aldrich, catalog number: P8340); store at -20 °C in aliquots to avoid freeze-thaw cycles

8. Tris-HCl (Sigma-Aldrich, catalog number: T5941)

9. Ammonium bicarbonate (ABC) (Sigma-Aldrich, catalog number: 09830)

10. Sequencing-grade trypsin (Promega, catalog number: V5111); store at -80 °C; reconstituted enzyme should be used immediately or stored at -20 °C for short-term use

11. Lys-C (Fujifilm Wako, catalog number: 125-05061); store at -80 °C

12. Formic acid (FA) (Fisher Scientific, catalog number: A117-50)

13. Indexed Retention Time (iRT) peptides (Biognosys, catalog number: Ki3002)

14. Phosphate-buffered saline (PBS) (Thermo Fisher Scientific, catalog number: 10010002)

**Solutions**

1. Lysis buffer for the bulk sample (see Recipes)

2. Lysis buffer for microdissected strips (see Recipes)

3. Digestion buffer for the bulk sample (see Recipes)

4. Digestion buffer for microdissected strips (see Recipes)

5. Buffer A (see Recipes)

6. Buffer B (see Recipes)

**Recipes**

**1. Lysis buffer for the bulk sample**

**Table:** 

Reagent	Final concentration	Quantity or volume
Urea	8 M	480 mg
SDC	1%	50 mg
TCEP	10 mM	100 μL
CAA	40 mM	100 μL
PIC	1×	100 μL
Tris-HCl (1 M, pH 8.5)	100 mM	500 μL
Total	n/a	To 1 mL

**2. Lysis buffer for microdissected strips**

**Table:** 

Reagent	Final concentration	Quantity or volume
SDC	1%	50 mg
TCEP	10 mM	100 μL
CAA	40 mM	100 μL
PIC	1×	100 μL
Tris-HCl (1 M, pH 8.5)	50 mM	500 μL
Total	n/a	To 1 mL

**3. Digestion buffer for the bulk sample**

Add Lys-C and trypsin in 100 mM ABC at an enzyme/substrate ratio of 50:1 (wt/wt) and incubate in a thermomixer at 37 °C at 1,000 rpm for 16 h.

**4. Digestion buffer for microdissected strips**

Digest in 50 mM ABC (pH 8.0) with an enzyme usage of 0.05 pg of trypsin per cubic micrometer tissue (pg/μm^3^).

**5. Buffer A**

0.1% FA in water

**6. Buffer B**

80% ACN with 0.1% FA

**Laboratory supplies**

1. Ceramic beads (1 and 3 mm) (MP Biomedicals, catalog numbers: 150010 and 150030)

2. Protein LoBind tubes (Eppendorf, catalog number: 0030108116)

3. 0.5 mL centrifuge tubes (Corning, catalog number: 3208)

4. 1.5 mL centrifuge tubes (Corning, catalog number: 3620)

5. 10 μL pipette tips (Axygen, catalog number: T-300-L)

6. 200 μL pipette tips (Axygen, catalog number: T-200-C-L)

7. 1,000 μL pipette tips (Axygen, catalog number: T-1,000-C)

8. Cell culture plates (Nunc EasyDish, catalog number: 150468)

9. C18 solid phase extraction disks (3M Empore, catalog number: 98060402173)

10. PEN membrane slides (Carl Zeiss, catalog number: 415190-9041-000)

11. Opaque adhesive caps (Carl Zeiss, catalog number: 415190-9181-000)

12. Ex kit (Omicsolution, catalog number: OSFP004-96)

## Equipment

1. Cryostat (Leica, model: CM1950)

2. Laser microdissection system (Leica, model: LMD7000)

3. Omni Bead Ruptor Elite homogenizer (OMNI)

4. Sonicator Chromatin and DNA Shearing System (Qsonica, catalog number: Q800R3)

5. Thermomixer (Eppendorf, catalog number: 5382000074)

6. Centrifuge 5424R (Eppendorf, catalog number: 5404000090)

7. Research^®^ Plus pipettes (0.1–2.5 μL) (Eppendorf, catalog number: 3123000217)

8. Research^®^ Plus pipettes (2–20 μL) (Eppendorf, catalog number: 3123000292)

9. Research^®^ Plus pipettes (20–200 μL) (Eppendorf, catalog number: 3123000250)

10. Research^®^ Plus pipettes (100–1,000 μL) (Eppendorf, catalog number: 3123000268)

11. SpeedVac concentrator (Thermo Fisher Scientific, catalog number: SPD1030A-230)

12. HPLC system (RIGOL, model: RIGOL L-3120)

13. Ultimate 3000 nanoLC system (Thermo Fisher Scientific)

14. timsTOF Pro mass spectrometer (Bruker Daltonics)

## Software and datasets

1. Data processing: Spectronaut, 16.2, commercial, paid

2. Deep learning: PyTorch, 1.13.1, free

3. Programming: Python, 3.9.13, free

4. Database: Uniprot/Mus musculus, 2022_01, free

5. Data repository: iProx, free

*Note: As an alternative to Spectronaut, users may consider DIA-NN (open-source) for DIA data processing, though parameter optimization may be required.*

## Procedure

**A. Tissue preparation and sectioning**

1. Take the brain of a C57BL/6J mouse according to approved ethical guidelines.

2. Rinse the brain with ice-cold PBS and embed it in OCT compound.

3. Snap-freeze the embedded brain in 2-methylbutane pre-cooled with liquid nitrogen.

4. Store frozen blocks at -80 °C until sectioning.

*Note: All steps should be performed on ice or at 4 °C to prevent protein degradation.*

5. Cut the frozen block into a series of 10-μm-thick horizontal sections using a cryostat.

*Note: Pre-cool the frozen block at -20 °C for 1 h and pre-cool the cryostat machine to a temperature of -20 °C to -30 °C.*

6. Collect sections on PEN membrane slides.

7. Store slides at -80 °C until microdissection.

**B. Laser microdissection**

1. Set up the Leica LMD7000 system according to the manufacturer’s instructions.

2. Imagine the whole-brain slide using the microscope camera.

3. For S4P sampling, select eight adjacent tissue slices.

4. Define two feature points in the brain tissue slice for angle alignment across adjacent slices.

*Note: Feature points should be clearly identifiable anatomical landmarks.*

5. For each slice, set a different cutting direction with 22.5° variation between adjacent slices.

*Note: The 22.5° increment (8 angles) ensures sufficient angular coverage for accurate tomographic reconstruction, which can be adjusted as needed; improvements in reconstruction algorithms can also reduce the requirement for angles.*

6. Program the LMD system to cut parallel strips of 300 μm width.

7. Collect dissected strips in 200 μL of opaque adhesive caps.

8. Verify successful sample collection under the microscope.

**C. Proteomic sample preparation (bulk sample)**

1. Add lysis buffer for the bulk sample to a mouse tissue in 0.5-mL reinforced polypropylene tubes with 1 and 3 mm ceramic beads.

2. Homogenize the tissue using an OMNI Bead Ruptor Elite instrument (10 m/s, 2 cycles, 30 s on, 15 s off) and then put it in ice water.

3. Centrifuge at 12,000× *g* for 20 min at 4 °C and collect the supernatant into the protein LoBind tubes.

4. Add digestion buffer for the bulk sample and incubate at 37°C, shaking at 1,000 rpm for 16 h.

6. Acidify samples with formic acid to pH 2–3.

7. Purify peptides with self-made stage-tips with reverse-phase C18.

8. Dry samples in a SpeedVac and reconstitute in 0.1% formic acid.

*Note: For DIA library construction in this study, a comprehensive spectral library was generated using data-dependent acquisition (DDA) mode. In brief, peptides were prepared from a homogenized sample (mouse brain strips), followed by high-pH reversed-phase fractionation to increase proteome depth [20]. The resulting fractions were analyzed by LC–MS/MS to build a spectral library, which was subsequently used to interpret the DIA data acquired from the microdissected tissue strips.*

**D. Proteomic sample preparation (microdissected strips)**

1. Add 10 μL of lysis buffer to each cap containing tissue strips.

2. Invert and incubate for 2 h at room temperature.

3. Centrifuge at 5,000× *g* for 2 min to collect lysate.

4. Add digestion buffer for microdissected strips.

5. Purify peptides using the Ex kit according to the manufacturer’s instructions.

6. Spike with iRT peptides for retention time calibration.

7. Dry samples in a SpeedVac and reconstitute in 0.1% formic acid.

*Note: Digested peptides can be stored at -20 °C for up to one week.*

**E. LC–MS/MS analysis**

1. Configure the Ultimate 3000 nanoLC system with an analytical column (25 cm × 75 μm i.d.).

2. Set the flow rate to 300 nL/min: start at 4% buffer B and 96% buffer A, followed by a stepwise increase of buffer B to 28% in 40 min, 44% in 5 min, 90% in 3 min, and stabilize for 3 min; then, decrease to 4% in 3 min and stabilize for 7 min.

3. Operate timsTOF Pro in diaPASEF mode with 22 × 40 Th precursor isolation windows (m/z 249–1229).

4. Set collision energy ramp from 59 to 20 eV across the ion mobility range.

5. Inject 200 ng of peptides per sample in the “sliding window” order [20] or randomized order to minimize batch effects.

*Note: Further details can be found in the original work [20].*

**F. Data preprocessing**

1. Process raw DIA data in Spectronaut 16.2 using default settings.

2. Search against the *Mus musculus* Uniprot database (UP000000589).

3. Set carbamidomethylation as a fixed modification, with oxidation and N-terminal acetylation as variable modifications.

4. Apply a 1% FDR cutoff at the precursor and protein levels.

5. Normalize protein intensities by non-zero median values in each sample (strips data).

*Note: A preprocessed spectral library generated using Spectronaut is available for users who wish to skip raw data processing and proceed directly to spatial reconstruction (available via iProX repository: IPX0007383000).*

6. Save preprocessed protein expression data (CSV format: rows = proteins, columns = strip samples) and a design matrix indicating strip angles and positions.

**G. Spatial reconstruction with DeepS4P**

1. Install the required Python packages: torch==1.13.1, numpy, and scipy.

2. Clone the DeepS4P repository from GitHub at https://github.com/Ma-JC/DeepS4P.

3. Load preprocessed protein expression data and design matrix.

4. Configure neural network parameters:

Input layer: 446 nodes

Hidden layers: 2048 and 4096 nodes with ReLU activation

Output layer: 7330 nodes

5. Set loss function parameters:

Reconstruction weight: 1.0

L2 regularization (λ): 0.001

Sparsity weight (α): 0.01

Smoothness weight (β): 0.1

6. Train the model for 1000 epochs with the Adam optimizer.

7. Apply a Gaussian filter (σ = 1) for image smoothing and noise reduction.

8. Generate spatial distribution maps for each protein.

*Note: Specific parameters can be adjusted according to variations in sample setup and other conditions.*

9. Expected output is a 2D spatial map per protein (saved as .npy or image file).

## Validation of protocol

This protocol has been used and validated in the following research article:

Qin et al. [20]. In-depth and high-throughput spatial proteomics for whole-tissue slice profiling by deep learning-facilitated sparse sampling strategy. *Cell Discovery* (Figures 2–7, Supplementary figures S1–S15).

Validation data include a comparison with immunohistochemistry staining for known regional markers (Figure 3a, Supplementary Figure S9), consistency with the gridding-based spatial proteomics approach (Supplementary Figure S2), reconstruction accuracy for marker proteins and spatially co-localized proteins (Figure 4), and reproducibility across technical and biological replicates (Figure 2, Supplementary Figure S4).

## General notes and troubleshooting

**General notes**

1. The current protocol uses a 300 μm strip width, but this can be adjusted based on desired spatial resolution and available MS time.

2. Eight adjacent slices are used for reconstruction, but future algorithm improvements can reduce this requirement.

3. The method is particularly suitable for tissues with relatively homogeneous protein distribution along the z-axis.

4. For tissues with high spatial heterogeneity in the z-direction, careful slice selection is recommended.

5. Safety precautions: Wear laser safety goggles during LMD operation. Handle cryostat blades and chemicals (e.g., formic acid, chloroacetamide) with appropriate personal protective equipment. Follow institutional guidelines for chemical waste disposal.

**Troubleshooting**

**Problem 1:** Incomplete tissue strip collection during LMD.

Possible causes: Laser power too low or incorrect focus.

Solutions: Optimize laser power and focus settings. Verify collection by visual inspection.

**Problem 2:** Poor spatial reconstruction quality.

Possible causes: Insufficient strip sampling or large batch effects.

Solutions: Ensure adequate coverage of projection angles. Implement strict quality control and batch correction.

**Problem 3:** Low protein identification in strips.

Possible causes: Insufficient sample amount or digestion efficiency.

Solutions: Optimize lysis and digestion conditions. Increase strip width if necessary.

**Problem 4:** High missing data rates.

Possible causes: Limited sample amount or MS sensitivity issues.

Solutions: Use larger strip widths or improve sample preparation protocols.

## References

[1] Mund A., Brunner A. D. and Mann M. (2022). Unbiased spatial proteomics with single-cell resolution in tissues. Mol Cell. 82(12): 2335 2349 2349. 10.1016/j.molcel .2022.05.022 35714588

[2] Svensson V., Teichmann S. A. and Stegle O. (2018). SpatialDE: identification of spatially variable genes. Nat Methods. 15(5): 343 346 346. 10.1038/nmeth.4636 29553579 PMC6350895

[3] Palla G., Fischer D. S., Regev A. and Theis F. J. (2022). Spatial components of molecular tissue biology. Nat Biotechnol. 40(3): 308 318 318. 10.1038/s41587-021-01182-1 35132261

[4] Rao A., Barkley D., França G. S. and Yanai I. (2021). Exploring tissue architecture using spatial transcriptomics. Nature. 596(7871): 211 220 220. 10.1038/s41586-021-03634-9 34381231 PMC8475179

[5] Hu J., Schroeder A., Coleman K., Chen C., Auerbach B. J. and Li M. (2021). Statistical and machine learning methods for spatially resolved transcriptomics with histology. Comput Struct Biotechnol J. 19: 3829 3841 3841. 10.1016/j.csbj .2021.06.052 34285782 PMC8273359

[6] Ratz M., von Berlin L., Larsson L., Martin M., Westholm J. O., La Manno G., Lundeberg J. and Frisén J. (2022). Clonal relations in the mouse brain revealed by single-cell and spatial transcriptomics. Nat Neurosci. 25(3): 285 294 294. 10.1038/s41593-022-01011-x 35210624 PMC8904259

[7] Jiang Y. R., Zhu L., Cao L. R., Wu Q., Chen J. B., Wang Y., Wu J., Zhang T. Y., Wang Z. L., Guan Z. Y., .(2023). Simultaneous deep transcriptome and proteome profiling in a single mouse oocyte. Cell Rep. 42(11): 113455 10.1016/j.celrep .2023.113455 37976159

[8] Harnik Y., Buchauer L., Ben-Moshe S., Averbukh I., Levin Y., Savidor A., Eilam R., Moor A. E. and Itzkovitz S. (2021). Spatial discordances between mRNAs and proteins in the intestinal epithelium. Nat Metab. 3(12): 1680 1693 1693. 10.1038/s42255-021-00504-6 34931081

[9] Buccitelli C. and Selbach M. (2020). mRNAs, proteins and the emerging principles of gene expression control. Nat Rev Genet. 21(10): 630 644 644. 10.1038/s41576-020-0258-4 32709985

[10] Lam K. H. B., Leon A. J., Hui W., Lee S. E., Batruch I., Faust K., Klekner A., Hutóczki G., Koritzinsky M., Richer M., .(2022). Topographic mapping of the glioblastoma proteome reveals a triple-axis model of intra-tumoral heterogeneity. Nat Commun. 13(1): e1038/s41467–021–27667–w. 10.1038/s41467-021-27667-w PMC8748638 35013227

[11] Carlyle B. C., Kitchen R. R., Kanyo J. E., Voss E. Z., Pletikos M., Sousa A. M. M., Lam T. T., Gerstein M. B., Sestan N., Nairn A. C., .(2017). A multiregional proteomic survey of the postnatal human brain. Nat Neurosci. 20(12): 1787 1795 1795. 10.1038/s41593-017-0011-2 29184206 PMC5894337

[12] Hosogane T., Casanova R. and Bodenmiller B. (2023). DNA-barcoded signal amplification for imaging mass cytometry enables sensitive and highly multiplexed tissue imaging. Nat Methods. 20(9): 1304 1309 1309. 10.1038/s41592-023-01976-y 37653118 PMC10482679

[13] Piehowski P. D., Zhu Y., Bramer L. M., Stratton K. G., Zhao R., Orton D. J., Moore R. J., Yuan J., Mitchell H. D., Gao Y., .(2020). Automated mass spectrometry imaging of over 2000 proteins from tissue sections at 100-μm spatial resolution. Nat Commun. 11(1): 8 10.1038/s41467-019-13858-z 31911630 PMC6946663

[14] Li L., Sun C., Sun Y., Dong Z., Wu R., Sun X., Zhang H., Jiang W., Zhou Y., Cen X., .(2022). Spatially resolved proteomics via tissue expansion. Nat Commun. 13(1): 7242 10.1038/s41467-022-34824-2 36450705 PMC9712279

[15] Ma M., Huo S., Zhang M., Qian S., Zhu X., Pu J., Rasam S., Xue C., Shen S., An B., .(2022). In-depth mapping of protein localizations in whole tissue by micro-scaffold assisted spatial proteomics(MASP). Nat Commun. 13(1): 7736 10.1038/s41467-022-35367-2 36517484 PMC9751300

[16] Hu B., He R., Pang K., Wang G., Wang N., Zhu W., Sui X., Teng H., Liu T., Zhu J., .(2025). High-resolution spatially resolved proteomics of complex tissues based on microfluidics and transfer learning. Cell. 188(3): 734 748 748 .e22. 10.1016/j.cell .2024.12.023 39855194

[17] Mulvey C. M., Breckels L. M., Geladaki A., Britovšek N. K., Nightingale D. J. H., Christoforou A., Elzek M., Deery M. J., Gatto L., Lilley K. S., .(2017). Using hyperLOPIT to perform high-resolution mapping of the spatial proteome. Nat Protoc. 12(6): 1110 1135 1135. 10.1038/nprot.2017 .026 28471460

[18] Hu B., He R., Pang K., Wang G., Wang N., Zhu W., Sui X., Teng H., Liu T., Zhu J., .(2025). High-resolution spatially resolved proteomics of complex tissues based on microfluidics and transfer learning. Cell. 188(3): 734 748 748 .e22. 10.1016/j.cell .2024.12.023 39855194

[19] Bhatia H. S., Brunner A. D., Öztürk F., Kapoor S., Rong Z., Mai H., Thielert M., Ali M., Al-Maskari R., Paetzold J. C., .(2022). Spatial proteomics in three-dimensional intact specimens. Cell. 185(26): 5040 5058 5058 .e19. 10.1016/j.cell .2022.11.021 36563667

[20] Qin R., Ma J., He F. and Qin W. (2025). In-depth and high-throughput spatial proteomics for whole-tissue slice profiling by deep learning-facilitated sparse sampling strategy. Cell Discov. 11(1): 21 10.1038/s41421-024-00764-y 40064869 PMC11894098

